# Rapid differentiation of human pluripotent stem cells into functional neurons by mRNAs encoding transcription factors

**DOI:** 10.1038/srep42367

**Published:** 2017-02-13

**Authors:** Sravan Kumar Goparaju, Kazuhisa Kohda, Keiji Ibata, Atsumi Soma, Yukhi Nakatake, Tomohiko Akiyama, Shunichi Wakabayashi, Misako Matsushita, Miki Sakota, Hiromi Kimura, Michisuke Yuzaki, Shigeru B. H. Ko, Minoru S. H. Ko

**Affiliations:** 1 Department of Systems Medicine, Keio University School of Medicine, 35 Shinanomachi, Shinjuku, Tokyo 160-8582, Japan; 2 Department of Physiology, Keio University School of Medicine, 35 Shinanomachi, Shinjuku, Tokyo 160-8582, Japan

## Abstract

Efficient differentiation of human pluripotent stem cells (hPSCs) into neurons is paramount for disease modeling, drug screening, and cell transplantation therapy in regenerative medicine. In this manuscript, we report the capability of five transcription factors (TFs) toward this aim: NEUROG1, NEUROG2, NEUROG3, NEUROD1, and NEUROD2. In contrast to previous methods that have shortcomings in their speed and efficiency, a cocktail of these TFs as synthetic mRNAs can differentiate hPSCs into neurons in 7 days, judged by calcium imaging and electrophysiology. They exhibit motor neuron phenotypes based on immunostaining. These results indicate the establishment of a novel method for rapid, efficient, and footprint-free differentiation of functional neurons from hPSCs.

Nervous system development and neural cell differentiation have been studied extensively, providing a foundation for current protocols of *in vitro* generation of various neuronal cell types from pluripotent stem cells and somatic cells^1^,^2^. Current neuronal cell differentiation protocols go step-by-step through a series of stages in order to generate neurons^3^. Typically, pluripotent stem cell (PSC) differentiation involves induction of neuroectoderm and then neural stem cell (NSC) formation through the inhibition of the bone morphogenetic protein (BMP) and Activin/TGFβ signaling pathways^4^,^5^. The multipotent NSCs are then directed to specific lineages, followed by terminal differentiation into post-mitotic neurons by a combination of patterning molecular cues, such as small molecules and growth/differentiation factors in the cell culture media. Although these procedures have been well established and used extensively, they have fundamental limitations in both speed and scale. The processes involved are relatively slow and typically take a few weeks before the formation of functional neurons. Complex, multi-step procedures - including the formation of embryoid bodies – obstruct the production of large quantities of homogenous cells, particularly for drug screening.

Alternatively, PSCs can also be rapidly and efficiently differentiated by inducing or overexpressing transcription factors (TFs) by plasmids, viruses, and other vectors^6^,^7^,^8^. However, these methods are associated with the potential problem of host genome modifications due to integration of foreign DNA into the genome.

Our group recently reported the differentiation of mouse embryonic stem (ES) cells into cells of different lineages such as myocytes, hepatocytes, blood cells, and neurons by introducing synthetic mRNA (syn-mRNA) encoding TFs^9^. Synthetic mRNA-based approaches have many desirable features for cell differentiation strategies. Unlike plasmids and some viruses, mRNAs do not integrate into the cell genome, achieving footprint-free gene product delivery. mRNA gets translated quickly upon entry into cells, and multiple rounds of translation lead to high levels of expression in a short time. In addition, dosage of synthetic mRNA is easily controlled, compared to dosage in viral transfection. Finally, multiple mRNA transcripts can be transfected together as a cocktail at the time of transfection^10^,^11^,^12^. Synthetic mRNAs allow for robust, footprint-free, and dose-dependent expression of any protein, making them ideal for use in cell differentiation.

In this paper, we describe the rapid differentiation of human pluripotent stem cells (both iPS and ES cells) using a synthetic mRNA cocktail consisting of proneural and neural differentiation TFs of the Neurogenin (NGN) and NeuroD (ND) families. Indeed, functional neurons accompanied with repetitive action potentials and calcium transients could be generated in a week after introduction of mRNAs into the cells. This new approach for rapid neurogenesis may prove to be useful for a variety of applications including disease modeling, drug screening, and possible cell transplantation therapy.

## Results

### Screening of candidate TFs

We inferred which candidate TFs to use based on our human gene expression correlation matrix (unpublished), which was generated essentially in the same manner as the mouse gene expression correlation matrix^13^,^14^,^15^. We selected the following TFs for multiple reasons: ASCL1, NEUROGENIN1 (NGN1), NEUROGENIN2 (NGN2), NEUROGENIN3 (NGN3), NEUROD1 (ND1), NEUROD2 (ND2), NEUROD4 (ND4), NEUROD6 (ND6), ATOH1, and ATOH7. These are all members of the basic-helix-loop-helix family of TFs, many of which have strong proneural effects^16^. In an earlier study, we showed that mouse Ascl1 is a strong inducer of neurons in mouse ESCs^9^. Neurogenins and NeuroDs are well-known master regulators of neurogenesis^16^,^17^. Plasmid- and viral-based NGN2 expression systems rapidly induced neurogenesis in several previous studies^6^,^7^,^8^,^18^,^19^. Recently, a polycistronic plasmid expression vector containing both NGN1 and NGN2 was shown to rapidly induce neurogenesis^6^.

**Figure 1:**
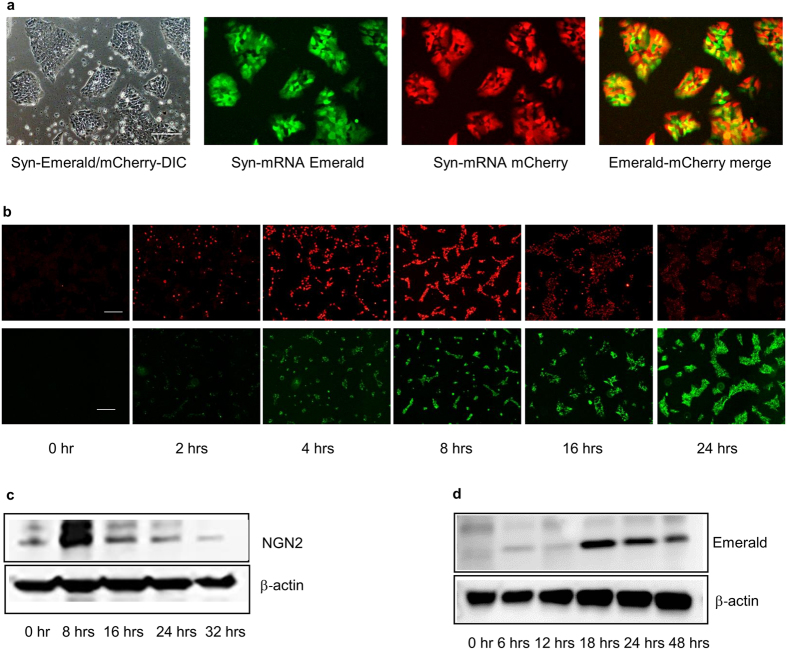
Delivery of syn-mRNAs into hPSCs. (**a**) Expression of synthetic messenger RNAs for fluorescent proteins Emerald and mCherry in TkDA3-4 human iPS cells 24 hours after transfection. Scale bar is 200 μm. (**b**) Kinetics of NGN2 (top panel) and emerald (bottom panel) synthetic mRNA expression in SEES3 human ES cells. Scale bars indicate 200 μm. (**c**) Time course of NGN2 protein expression in lysates of human ES cells transfected with syn-NGN2 mRNA. Cropped images of the immublot corresponding NGN2 and B-actin protein bands are shown. Original blots are included in Supplementary Figures S3a and b. (**d**) Time course of Emerald protein expression in lysates of human ES cells transfected with syn-Emerald mRNA. β-actin was used as a loading control. Cropped images of the immunoblot corresponding Emerald and β-actin protein bands are shown. Original blots are included in Supplementary Figures S4a and b.

We examined whether these TFs can differentiate human pluripotent stem cells (hPSCs) into neural cell types. To develop a footprint-free cell differentiation protocol, we explored the use of synthetic mRNAs (syn-mRNAs) to deliver TFs to hPSCs. First, synthetic mRNAs encoding green fluorescence protein variants — Emerald (syn-Emerald) and mCherry (syn-mCherry) were transfected into TkDA3-4 induced pluripotent stem cells (iPSCs). Their expression was monitored by fluorescence microscopy (Fig. 1a). We confirmed efficient production of the proteins in these cells as well as co-expression of both proteins in most of the cells (Fig. 1a). We also examined the kinetics of protein production from transfected syn-mRNAs by using syn-NGN2 as a representative TF and fluorescent protein marker syn-Emerald as a control. Production of NGN2 protein after the introduction of syn-NGN2 mRNA into SEES3 human embryonic stem cells (hESCs)^20^ was detected as early as 2 hours after transfection, peaked at around 8 hours, and declined precipitously thereafter (Fig. 1b, upper panel). By contrast, a control Emerald protein continued to be expressed over 24 hours after transfection (Fig. 1b, lower panel). Western-blotting analysis of NGN2-transfected human ES cell lysates also revealed strong expression of NGN2 protein by 8 hours and a rapid decline thereafter (Fig. 1c), whereas the expression of Emerald protein continued to increase up to and beyond 24 hours after transfection (Fig. 1d). The transient nature of TF protein expression was similar to previously published reports^10^,^11^

Analysis of the induction kinetics of the NGN2 protein in TkDA3-4 iPS cells revealed a very rapid induction as early as 30 minutes after transfection, which continued up to 8 hours (Supplementary Figure S1). Around 80% of the cells stained positive for NGN2 protein expression by 90 minutes after transfection.

**Figure 2:**
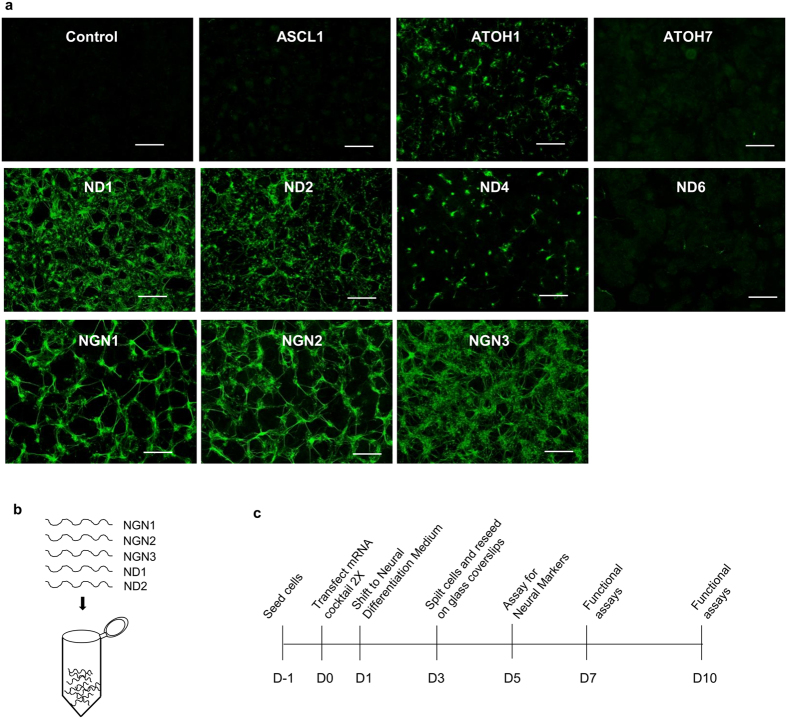
Induction of neurogenesis by syn-TFs mRNAs of Neurogenin and NeuroD families. (**a**) Neuronal differentiation induced by synthetic mRNAs for different Neurogenins and NeuroD TFs in TkDA3-4 human iPS cells (assayed at Day 5). For control, syn-Emerald and syn-mCherry were transfected. Expression of neuron-specific βIII-Tubulin (TUBB3) was measured by immunocytochemistry. Scale bars indicate 500 μm. (**b**) Syn-5TFs cocktail contains equal amount (μg) of NGN1, NGN2, NGN3, ND1 and ND2 syn-mRNAs. (**c**) Experimental protocol for the generation of motor neurons from pluripotent stem cells. From Day −1 (D-1) to Day 10 (D10).

We then transfected each of the syn-mRNAs encoding ASCL1, NGN1, NGN2, NGN3, ND1, ND2, ND4, ND6, ATOH1, and ATOH7 into TkDA3-4 human iPS cells. Judging by the immunostaining of neuron-specific tubulin beta-3 chain (TUBB3) and cell morphologies five days after the introduction of syn-mRNAs, ASCL1, ND4, ND6, and ATOH7 showed only a mild neural induction, whereas ATOH1 induced a moderate expression of neuron-specific TUBB3. Syn-mRNAs encoding NGN1, NGN2, NGN3, ND1, and ND2 showed a strong neural induction by Day 5 (Fig. 2a). The same five factors — NGN1, NGN2, NGN3, ND1, and ND2 — efficiently induced the differentiation of human ES cells into neurons (Supplementary Figure S2). Therefore, we decided to focus on these five TFs for the following studies.

### A cocktail of five TFs efficiently induces neurogenesis

NDs are downstream effectors of NGNs^21^,^22^. Also, ectopic expression of NGNs can induce both neural and glial differentiation, whereas NGN-mediated ND expression causes the terminal differentiation of neurons^21^,^22^. Therefore, we reasoned that combining both NGNs and their effectors, i.e., NDs, can enhance the speed and efficiency of neuronal differentiation of PSCs. Because syn-mRNAs can be mixed in any combination, we were able to test quickly cocktails of syn-mRNAs encoding NGN1, NGN2, NGN3, ND1, and ND2, called “syn-5TFs” (Fig. 2b). Because the protein expression of the syn-mRNAs is transient and peaks at around 8 hours before rapidly declining, we optimized transfection conditions and found that two transfections of the syn-5TFs cocktail were sufficient to induce neural differentiation of PSCs as depicted in Fig. 2c.

**Figure 3:**
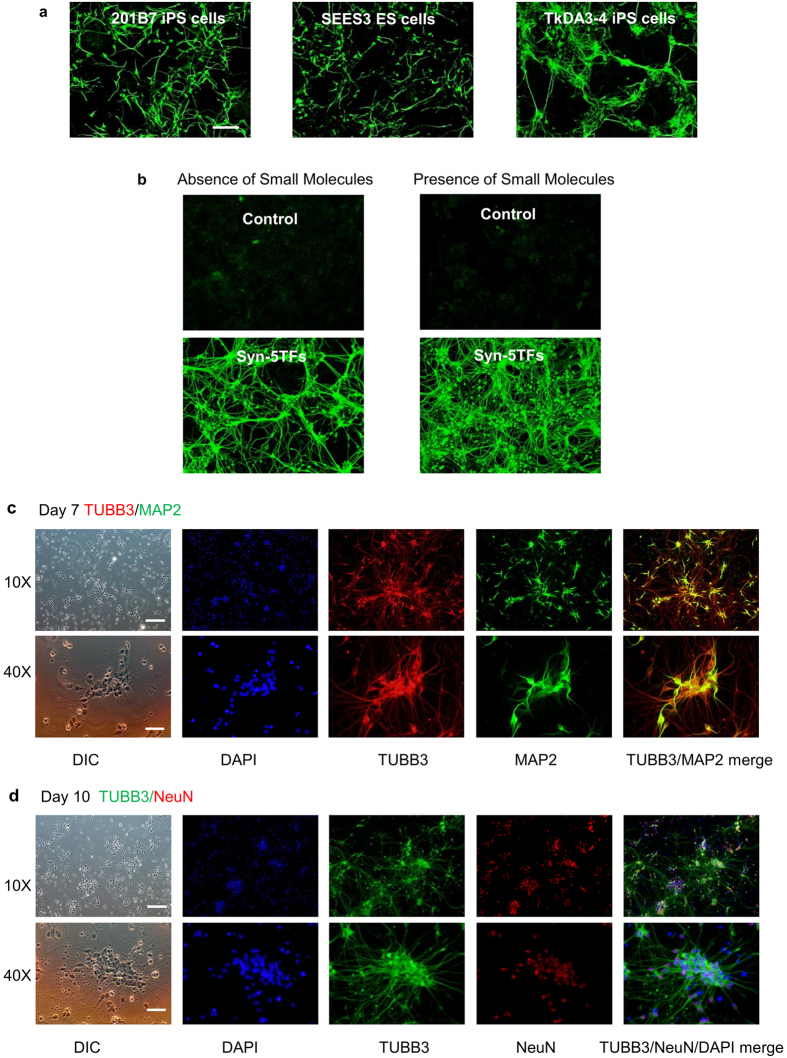
Induction of neurogenesis in human pluripotent stem cells by syn-5TFs mRNA cocktail. (**a**) Syn-5TFs-induces the efficient differentiation of human ES (SEES3) and iPS (201B7 and TkDA3-4) lines into neuronal cells. Representative images of TUBB3 staining at Day 5 are shown. The efficiency of TUBB3 expression in ES and iPS lines were as follows: TkDA3-4 human iPS cells, 86.2% ± 2.2 [mean ± SD] (two independent experiments, n = 2); 201B7 human iPS cells, 89.2% ± 9.6 [mean ± SD] (n = 2); and SEES3 human ES cells, 88.0% ± 12.1 [mean ± SD] (n = 2). Scale bar indicates 200 μm. (**b**) Small molecule modulators enhance the efficiency of neuronal differentiation induced by the Syn-5TFs cocktail in TkDA3-4 human iPS cells. Representative images of TUBB3 staining at Day 5 are shown. Scale bar indicates 200 μm. (**c**) Highly efficient neuronal differentiation of TkDA3-4 human iPS by Syn-5TFs cocktail. Neuronal TUBB3 (red) and MAP2 (green) expressing cells at Day 7 are shown. Upper row shows images of low magnification (10X). Scale bar indicates 200 μm. Lower row shows images of high magnification (40X). Scale bar indicates 50 μm. The fraction of MAP2-positive cells among TUBB3-positive neurons (at Day 7) was 83.2% ± 0.6 [mean ± SD] (n = 2). (**d**) Highly efficient neuronal differentiation of TkDA3-4 human iPS by Syn-5TFs cocktail. Neuronal TUBB3 (green) and NeuN (red) expressing cells at Day 10 are shown. The fraction of NeuN-positive cells among TUBB3-positive neurons (at Day 10) was 96.3% ± 0.4 [mean ± SD] (n = 2). Upper row shows images of low magnification (10X). Scale bar indicates 200 μm. Lower row shows images of high magnification (40X). Scale bar indicates 50 μm.

When we transfected both ESCs (SEES3) and iPSCs (TkDA3-4 and 201B7) with the syn-5TFs cocktail, we found that rapid and highly efficient neural differentiation occurred by Day 5 after transfection (Fig. 3a). Efficiency of neural differentiation was similar between ESCs and iPSCs judged by the number of cells marked with neuron-specific TUBB3 in the total number of cells in the culture: 86.2% ± 2.2 [mean ± SD] (two independent experiments, n = 2) for TKDA3-4 human iPS cells, 89.2% ± 9.6 [mean ± SD] (n = 2) for 201B7 human iPS cells, and 88.0% ± 12.1 [mean ± SD] (n = 2) for SEES3 human ES cells. We did not notice any difference in the kinetics of differentiation between the cell lines (data not shown).

We further found that adding well-known neural modulating small molecules — retinoic acid (included in the B27 supplement added to the differentiation medium), forskolin, SB431542, and dorsomorphin — to the neural differentiation medium^18^ greatly enhanced the efficiency of neural differentiation initiated by the syn-5TFs cocktail (Fig. 3b). This suggests that, for an efficient neuronal differentiation, both cell-intrinsic signals mediated by TFs and extracellular differentiation cues from small molecules are important. On the other hand, the small molecules alone had no effect on neurogenesis when used without TFs (Fig. 3b). Therefore, in all further experiments, we used the syn-5TFs cocktail to initiate neuronal differentiation and added a small molecule cocktail consisting of forskolin, SB431542, dorsomorphin, retinoic acid in N2B27 neural culture medium (Fig. 2c).

### Characterization of syn-5TFs-induced neurogenesis

Immunocytochemistry of syn-5TFs-transfected TkDA3-4 iPS cells on Day 7 revealed that over 95% of cells (98.2% ± 2.3 [mean ± SD]: seven independent experiments, n = 7) were positive for neuron-specific TUBB3 (Fig. 3c). Further immuno-cytochemical analyses revealed that by Day 7 around 80% (83.2% ± 0.6 [mean ± SD], n = 2) of the TUBB3-positive neurons were already positive for a microtubule-associated protein 2 (MAP2), a relatively mature neuronal marker (Fig. 3c, top panels). By Day 10, the mature neuronal differentiation marker, NeuN, was detected in around 95% of the TUBB3-positive neurons (96.3% ± 0.4 [mean ± SD], n = 2) (Fig. 3c, bottom panels).

**Figure 4:**
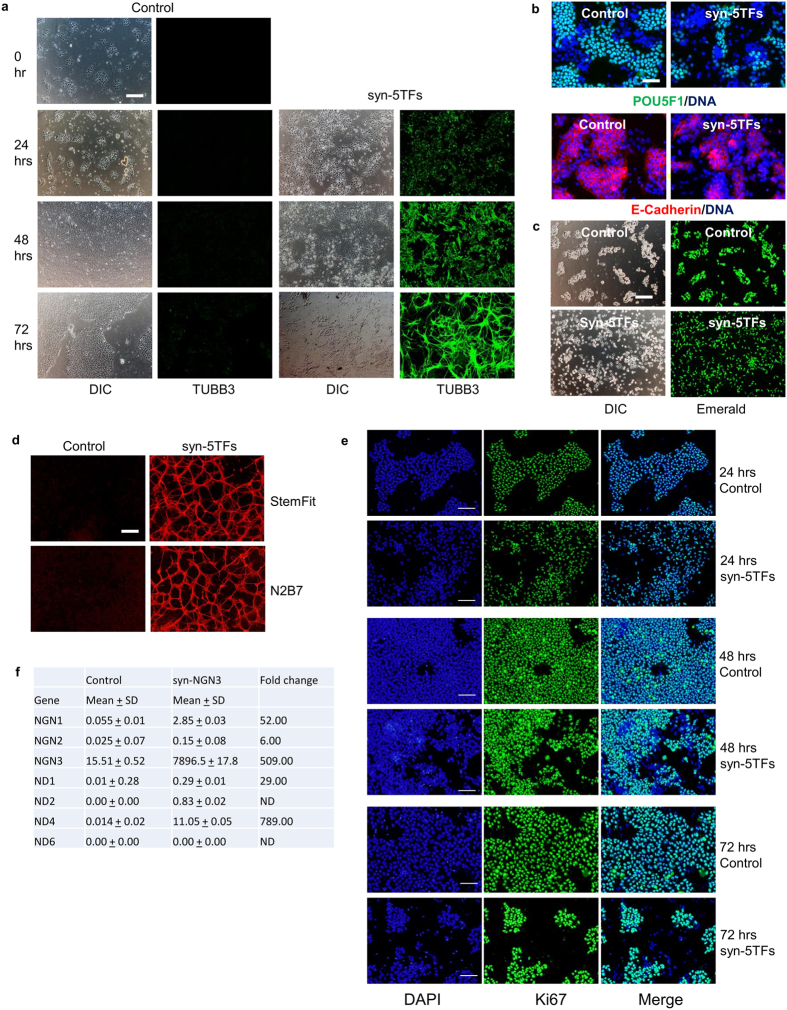
Kinetics and morphological changes during neuronal differentiation induced by the syn-5TFs cocktail. (**a**) Kinetics of neuronal TUBB3 expression during syn-5TFs mRNA cocktail-induced TkDA3-4 iPS cell differentiation. Scale bar indicates 200 μm. (**b**) Expression of pluripotent markers 24 hours after the induction of differentiation of TkDA3-4 human iPS cells by syn-5TFs cocktail. Scale bar indicates 200 μm. (**c**) Morphological changes during syn-5TFs-induced differentiation of human TkDA3-4 iPS cells. Control cells were transfected with syn-Emerald. syn-5TFs-transfected cells were also cotransfected with syn-Emerald to visualize cells. Scale bar indicates 200 μm. (**d**) Syn-5TFs mRNA cocktail induces differentiation of human TkDA3-4 iPS cells even in the pluripotency-sustaining condition (in the StemFit culture media). The differentiation-promoting condition (in the N2B27 culture media) is also shown for comparison. Representative images of TUBB3 staining are shown (at Day 4). Scale bar indicates 100 μm. (**e**) Analyses of iPS cell proliferation during syn-5TFs-induced differentiation. Scale bar indicates 200 μm. Nuclei (blue) and Ki67 (green). (**f**) ectopic expression of syn-NGN3 TF activates the expression of other pro-neural TF expression. Measurements were done by RNA-sequencing. FPKM scores and fold changes are shown. ND = not determined.

Next, we characterized the events occurring during neuronal differentiation of PSC mediated by the syn-5TFs cocktail. Time course analyses of neuronal-specific TUBB3 expression revealed that syn-5TFs-transfected TkDA3-4 iPSCs started to express the TUBB3 marker as early as 24 hours after transfection and continued to increase over three days (Fig. 4a). Similar results were obtained with SEES3 human ES cells (data not shown).

During the differentiation phase, the expression of pluripotency markers such as POU5F1 (also known as OCT4 or OCT3/4) and E-cadherin proteins decreased dramatically within 24 hours of the induction of differentiation by the syn-5TFs when assayed by immunostaining (Fig. 4b). Morphologically, cells undergoing neuronal differentiation started to delaminate from the ES/iPS colonies by 8 hours after the introduction of the syn-5TFs cocktail. By 24 hours, they largely flattened out and existed mostly as single cells, unlike PSC which still continued to grow in colonies (Fig. 4c). Further analysis of the differentiation events revealed that syn-5TFs-transfected cells underwent neuronal differentiation even in pluripotency-sustaining culture conditions (see Methods). The iPS cells differentiated with similar efficacy in both StemFit medium as well as in the N2B27 neural differentiation medium (Fig. 4d). This suggests that intrinsic TF signaling overrides extrinsic pluripotency-sustaining cues for neuronal differentiation. This observation seems to be consistent with an earlier report that the NGN2-induced differentiation of human ES cells into mature neurons also occurs under pluripotency-sustaining conditions^19^.

Treatment with the syn-5TFs cocktail also decreased cell proliferation, which was demonstrated by the decreased expression of a proliferative cell-marker, Ki67. Downregulation of Ki67 in iPSCs by the syn-5TFs was observed as early as 24 hours after transfection. By Day 3, a roughly 40% reduction was observed compared to untreated cells (Fig. 4e). These results are in line with reports that ectopic expression of NGNs leads to rapid cell cycle withdrawal during neuronal differentiation^23^.

We next checked whether the expression of a member of the syn-5TFs cocktail is capable of inducing the expression of other TFs included in the syn-5TFs cocktail. Using syn-NGN3 as a representative TF, we found that its expression in human ESCs strongly induced the expression of other TFs of the cocktail (Fig. 4f). These results suggest that along with multipronged differentiation induced by NGNs and NDs, a cross activation of expression among these factors is likely the reason for the rapid induction and generation of neurons we observed in this study.

### Syn-5TFs-induced neurons exhibit stimulus-induced channel activities and electrical properties

**Figure 5:**
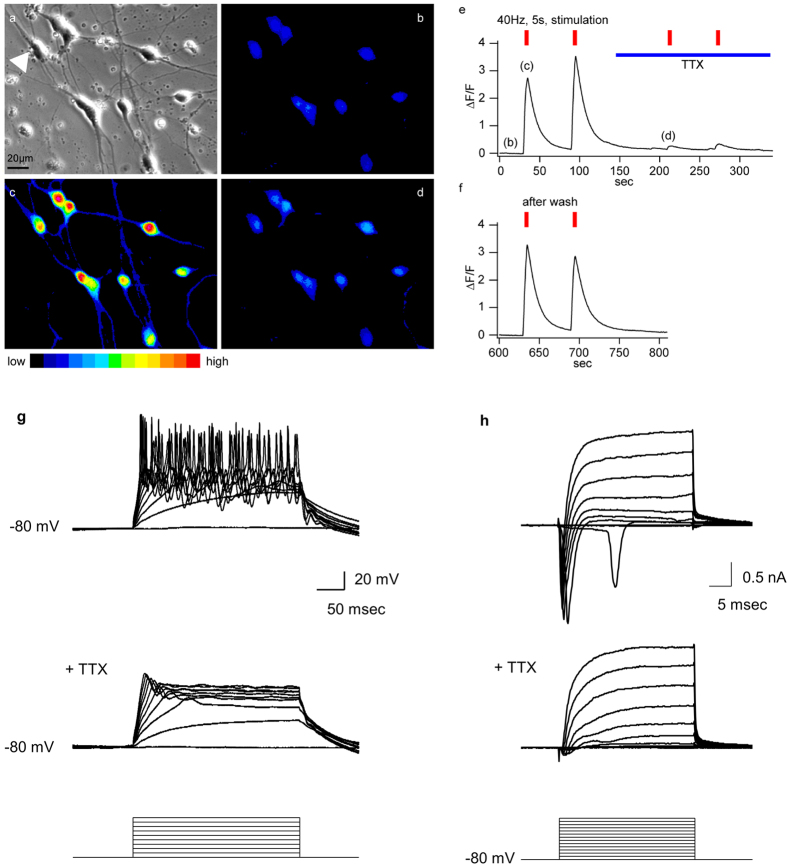
Syn-5TFs-induced neurons are functional. (**a–f**) Electric stimulation-evoked calcium transients in neurons induced by syn-5TFs on Day 7. Fluo-4 loaded cells before electric stimulation in DIC microscopy (**a**) and fluorescence microscopy (**b**). Cells after electric stimulation with 40 Hz pulses for 5 secs (**c**). Cells pre-treated with tetrodotoxin (TTX) (0.5 μM) for two min to block sodium channels, followed by electric stimulation (**d**). (**e**) Time line of representative trace of cell, indicated by an arrowhead in (**a**). The timings of image capture are indicated as (**b–d**). (**f**) Time line trace shows the recovery of cells to evoke calcium transients after TTX wash-off. (**g**) Repetitive multiple action potentials induced by current injections in the current clamp mode in syn-5TFs-induced neurons on Day 10. Application of TTX blocked the firing. (**h**) In a voltage clamp mode, syn-5TFs-induced neurons exhibited fast activating and inactivating sodium currents, which were blocked by TTX. The square pulses shown in the bottom panels indicate 20 pA current steps in (**g**) or 10 mV voltage steps in (**h**) applied to the syn-5TFs-induced neurons.

We then functionally characterized the syn-5TFs-derived neurons by Fluo-4-based Ca^2+^ imaging. Electric field stimulation at 40 Hz for 5 seconds (secs) induced Ca^2+^ increase in most syn-5TFs-derived neurons from TkDA3-4 iPS cells by Day 7 (94%, n = 89; Fig. 5a–d and Supplementary Movie M1). Addition of tetrodotoxin (TTX), a neuronal voltage-gated sodium channel blocker, to the extracellular solution reversibly inhibited the electric stimulation-induced Ca^2+^ increase (Fig. 5e,f). These data indicate that these Ca^2+^ transients were mediated by voltage-gated sodium channels, followed by Ca^2+^ influx through voltage-gated Ca^2+^ channels.

The gold standard of functional neurons is their ability to exhibit action potentials. At Day 7, whole-cell patch-clamp recordings revealed that approximately half of the neurons derived from iPS cells (TkDA3-4 iPSCs) showed a single action potential during 300-ms depolarization in a current-clamp mode. By Day 10, however, most iPSC-derived neurons (94%, n = 35 cells) showed action potentials (Supplementary Table 3). Furthermore, approximately 60% of the cells (21 out of 35) exhibited repetitive action potentials, which were blocked by TTX (Fig. 5g). Voltage-clamp recordings further confirmed TTX-sensitive fast activating and inactivating inward currents induced by depolarizing voltage pulses in iPSC-derived neurons (Fig. 5h). Because generation of action potentials requires high density of voltage-gated sodium channels^24^, these findings indicate that maturation of iPSC-derived neurons proceeded significantly during additional 3-day culture from Day 7. These results indicate that the Syn-5TFs cocktail rapidly differentiate iPSCs into functional neurons *in vitro*.

### Syn-5TFs mRNA cocktail generates cells expressing motor neuron markers

**Figure 6:**
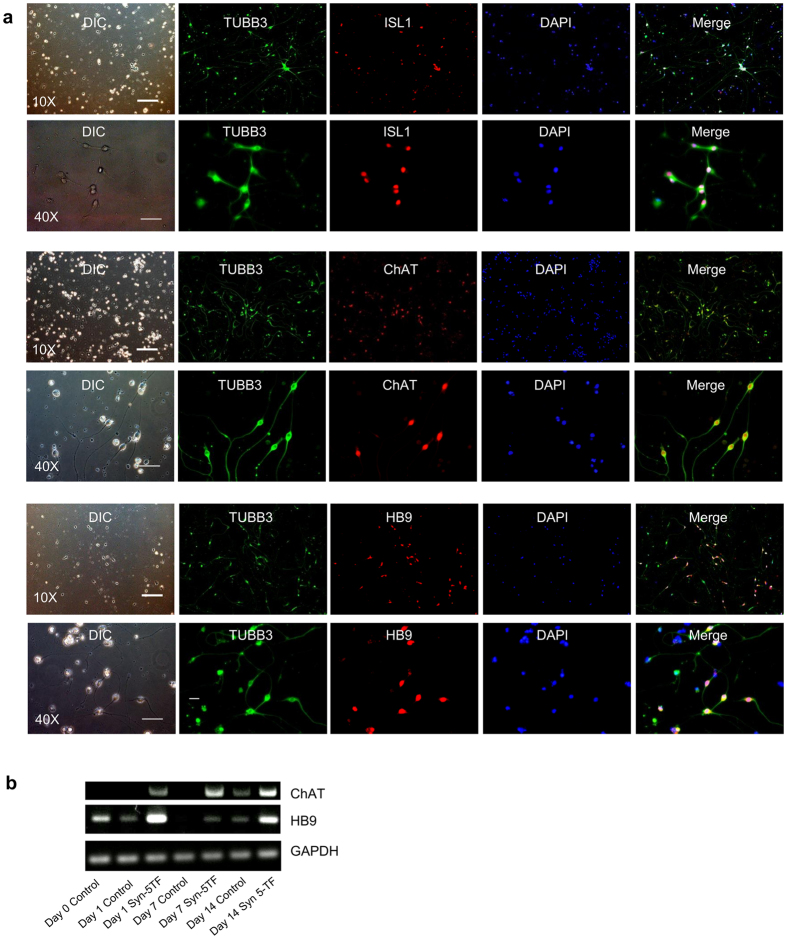
Syn-5TFs-induced motor neurons and their functional characterization. (**a**) Syn-5TFs differentiate human iPS cells into motor neurons. Day 7 neurons stained with ISL1 (top two panels), ChAT (middle two panels), and HB9 (bottom two panels). Upper row of each panel shows low magnification images. Scale bar indicates 200 μm. Lower row of each panel shows higher magnification images. Scale bar indicates 50 μm. The fractions of marker-positive cells among TUBB3-positive neuronal cells were ISL1 (100% ± 0 [mean ± SD], two independent experiments, n = 2), ChAT (97.7% ± 2.28 [mean ± SD], n = 3), HB9 (94.3% ± 4.2 [mean ± SD], n = 3). (**b**) Expression of motor neuron markers analyzed by RT-PCR in syn-5TFs-induced neurons.

Next, we characterized the neuronal subtypes specified by the syn-5TFs cocktail. The expression of markers associated with the motor neuron phenotype started to appear by Day 7 and became apparent by Day 10 when functional neurons were obtained. By Day 7, iPS-derived neurons stained positively for the canonical motor neuron markers ISL1, choline acetyl transferase (ChAT), and motor neuron and pancreas homeobox protein 1 (also known as HB9) (Fig. 6a). Quantitative immunocytochemistry revealed that almost all the TUBB3-positive neuronal cells were stained positively for HB9 (94.3% ± 4.2 [mean ± SD], three independent experiments, n = 3), ChAT (97.7% ± 2.28 [mean ± SD], n = 3), and ISL1 (100% ± 0 [mean ± SD], n = 2), indicating a high efficiency of differentiation toward motor neuron subtype. Reverse-transcription-PCR analysis revealed the induction of the motor neuron markers ISL1, HB9, and ChAT as early as 24 hours after transfection, suggesting that these genes might be direct targets of the syn-5TFs cocktail. By Day 7 and Day 14, the expression of these markers became apparent (Fig. 6b). Quantitative reverse transcription PCR of motor neuron associated genes showed enhancement of expression of MN-marker genes ISL1, HB9, ChAT and CHT-1, a high affinity choline transporter gene, during the course differentiation of iPS cells (Supplementary Figure S5). These results support earlier observations that NGNs can specify the motor neuron lineage^7^,^18^,^25^,^26^.

## Discussion

Collectively, our data shows a novel, rapid, and highly efficient way to derive functional neurons from pluripotent stem cells by using a syn-mRNA cocktail of 5 TFs. Compared to plasmid and viral vectors for gene delivery, syn-mRNAs have many advantages such as rapid and high levels of protein production due to multiple rounds of translation^11^,^27^, having the ability and option to combine multiple mRNAs, being footprint-free in delivery of TFs. Since mass-production of syn-mRNAs is possible through *in vitro* transcription, our method also has possibilities for scalability in the production of neurons as well.

Our study reveals the generation of motor neurons expressing canonical motor neuron markers such as ISL1, ChAT, and HB9. In our method, initiation of neuronal TUBB3 expression is much earlier than in previous reports, including rapid neuronal differentiation induced by NGN2^6^,^8^. Although NGNs and NDs are already potent neuronal inducing factors by themselves, a combination of them definitely makes it possible to achieve high efficiency and rapidity in developing motor neurons. We also noted that the addition of small molecule cues significantly enhanced the derivation of motor neurons. These small molecules included potent proneural-inducing molecules such as SMAD inhibitors dorsomorphin and SB431542^5^, retinoic acid - a strong modulator of neurogenesis, and forskolin - a cyclic AMP enhancer^28^. Indeed, these cells exhibited electric stimulation-induced Ca^2+^ transients by Day 7. Furthermore, by Day 10, most of these neurons showed repetitive action potentials — indicative of functional neurons.

The technology presented in this paper could also provide therapeutic interventions and methods of study for diseases characterized by loss of somatic motor neurons which control peripheral muscle targets. These diseases, such as amyotrophic lateral sclerosis (ALS) and spinal muscular atrophy, are often progressive and untreatable. Our efficient method to generate motor neurons *in vitro* from pluripotent stem cells such as ESCs and iPSCs could be used for drug screening to identify therapeutic compounds, disease modeling to further study the molecular mechanisms underlying these diseases, and cell replacement therapy in regenerative medicine. In addition, syn-mRNA’s lack of a footprint could help assuage fears of integration of virus and other foreign DNAs into the host genome that are typical of other delivery methods.

Our strategy can also be adapted to generate other cell types as well as neuronal lineage subtypes. Differentiating PSCs through use of syn-TFs may be a potent framework for generating various cell types *in vitro* for modeling difficult-to-study diseases, studying cellular signaling mechanisms, and possibly using them for cell replacement therapy in regenerative medicine.

## Methods

### Cells and culture conditions

SEES3 human ES cells^20^ were obtained from Dr. Hidenori Akutsu, TkDA3-4 human dermal fibroblast-derived iPS cells^29^ were obtained from Dr. Koji Eto. 201B7 human iPS cells were obtained from RIKEN Cell Bank. SEES3 human ES, TkDA3-4, and 201B7 human iPS cells were cultured under feeder-free conditions in StemFit AK03 medium (Ajinomoto, Tokyo, Japan) on laminin511 (iMatrix-511: Nippi, Tokyo, Japan)-coated dishes.

### The chemicals used and their suppliers

Recombinant Laminin 511-E8b fragments (iMatrix-511: Nippi, Tokyo, Japan). Dorsomorphin (EMD chemicals, Darmstadt, Germany). SB431542, Forskolin, Poly-L-Ornithine, Alpha-Bungarotoxin-tetramethyl rhodamine (Sigma-Aldrich, St. Louis, MO, USA). BDNF (PeproTech, Rocky Hill, NJ, USA). GDNF and NT3 (R&D systems, Minneapolis, MN, USA). DMEM/HAM F-12, Neurobasal medium, OPTIMEM, N2, and B27 supplements (Life Technologies, Carlsbad, CA, USA). Recombinant B18R protein (eBioscience-Affymetrix, San Diego, CA, USA). Modified nucleotides 5-methylcytidine-5′-triphospahte and pseudouridine-5′-triphosphate were obtained from Trilink biotechnologies (San Diego, CA, USA). KAPA HiFi Hot Start ready mix (Kapa Biosystems, Boston, MA, USA). MEGAscript T7 kit, MEGAclear kit, and RNA Millennium markers (Ambion, Thermo Fisher Scientific, Waltham, MA, USA). Anti-reverse cap analog 3′-O-Me-m7-G(5′)ppp(5′)G and Antarctic Phosphatase (New England Biolabs, Ipswich, MA, USA). 4% PFA-PBS (WAKO Pure Chemical Industries, Osaka, Japan), glass coverslips (13/15/18 mm) (Matsunami Glass IND, Osaka, Japan). 35 and 60 mm cell culture dishes (AGC techno glass, Shizuoka, Japan), Four-well plates (NUNC, Thermo Fisher Scientific, Waltham, MA, USA). Twenty-four-well plates (Costar, Sigma-Aldrich, St. Louis, MO, USA). PCR cleanup kit (Macherey-NAGEL, Duren, Germany). Trizol (Life Technologies, Carlsbad, CA, USA). Direct-zol RNA mini prep kit (Zymo Research). Total RNA from Trizol was extracted using the Direct-zol RNA mini prep kit (Zymo Research Corp., Carlsbad, CA, USA). cDNA was synthesized using the ReverTra Ace qPCR RT PCR Master mix (TOYOBO, Osaka, Japan). Reverse transcription PCR was performed using the Ex-Taq enzyme (Takara-Bio, Shiga, Japan). RT-PCR and quantitative RT-PCR primers used are listed in the Supplementary Table S2. Primary and secondary antibodies used are listed the Supplementary Table S3 and were obtained from the following suppliers: Cell Signal Technologies (CST, Danvers, MA, USA); Roche Diagnostics (Mannheim, Germany); DSHB (University of Iowa, Iowa City, USA); Abcam (Cambridge, UK); Sigma-Aldrich; Merck Millipore (Darmstadt, Germany); and Santa Cruz BioTechnologies (SCBT, Dallas, TX, USA).

### Generation of synthetic messenger RNAs

Using the LR reaction protocol (Thermo Fisher Scientific, USA), we cloned open reading frames (ORFs) of desired TFs into the PCR2-UTR-R1R2 vector. PCR2-UTR-B1B2 vector was linearized with a restriction enzyme that cuts outside the ORF. The linearized vector was used as the template in a tail PCR reaction which utilized a 5′ primer with a T7 polymerase promoter sequence and a 3′UTR directed primer with a long poly T tail. The PCR product was gel purified and was used as a template in an *in vitro* transcription (IVT) protocol essentially as described by Mandal and Rossi^11^. The IVT reaction included an anti-reverse cap analog (ARCA) and modified nucleotides, 5-methyl cytidine-5′-triphosphate and pseudouridine-5′-triphosphate, to reduce cytotoxicity due to the activation of innate immune responses. The purified synthetic mRNA obtained was size verified and stored as aliquots at −80 °C until use.

### Differentiation protocol

Pluripotent stem cells were seeded and cultured overnight in four-well plates at a density of 50,000–70,000 cells per well in StemFit AK03 medium containing Y27632. The next day, before mRNA transfection, the medium was replaced with StemFit AK03 containing B18R (200 ng/ml final concentration), a recombinant receptor that binds and neutralizes the type 1 interferons to prevent toxicity during mRNA transfection. The mRNA was transfected using Lipofectamine Messenger Max reagent according to manufacturer’s instruction. Briefly, 1 μg mRNA cocktail in OptiMEM was mixed with 2 μl messenger max in OptiMEM reduced Serum Media and incubated at room temperature for five minutes for complex formation. Complexes were then added dropwise to wells. Medium was replaced three hours after transfection with StemFit AK03 containing B18R. After a two-hour recovery period, the cells were transfected again as above for a second time. In some experiments, cells were transfected two more times the next day (total of four times). After overnight incubation after the final transfection, the medium was replaced with neural differentiation medium (1:1 mixture of DMEM/F12 HAM and Neurobasal medium with N2 and B27 supplements) containing small molecule cocktail dorsomorphin, SB431542, and forskolin all at a final concentration of 3.3 μM to enhance neuronal generation^18^. Two days after the start of transfection, the cells were passaged and cultured on ornithine/LM coated glass coverslips in the differentiation medium. The medium was replaced every day for five days. After that, the medium was replaced with N2B27 containing BDNF, GDNF, and NT-3 (all at 10 ng/ml). Half the medium was replaced every other day. In all the experiments (except one experiment shown in Fig. 4f, where cells were transfected four times with syn-NGN3), the cells were transfected twice with syn-5TFs mRNA cocktail. In our hand, four-time transfections slightly enhanced the efficiency but also increased cytotoxicity. We, therefore, routinely carried out transfection twice.

### Immunocytochemistry

The medium was removed from wells, and cells were rinsed with PBS once and fixed with 4% paraformaldehyde (Wako) at RT for 20 min. This was followed by permeabilization with 0.2% Triton-X-100 (in 5% BSA) for 12 min. After washing three times with PBS, the cells were blocked with either 5% BSA or 5% goat serum for 30 min at room temperature. The cells were then incubated with the primary antibodies overnight at 4 °C. After washing off the unbound primary antibodies, the cells were incubated for 45 to 60 min at room temperature with Alexa Fluor conjugated secondary antibodies. Cells were washed three times with PBS, and DAPI was added to stain the nucleus. Fluorescent labeling was verified, and the stained cells were photographed by Olympus microscope (IX73). After the staining process, the coverslips were mounted in VECTASHIELD Mounting Medium (Vector Laboratories). In some experiments, cells were photographed using a DeltaVision Elite microscope (GE Healthcare).

### Electrophysiology

Neurons derived from iPSCs transfected with syn-5TFs mRNAs were used for electrophysiological analyses on Day 7 and 10 as described previously^30^. Briefly, whole-cell current-clamp or voltage-clamp recordings were performed using Axopatch 200B (Axon Instruments, USA) at room temperature. Cells were continuously perfused with the extracellular solution composed of 117 mM NaCl, 2.5 mM KCl, 2 mM CaCl_2_, 2 mM MgCl_2_, 15 mM d-glucose and 20 mM HEPES (pH 7.4 adjusted with NaOH, 304 mOsm). Patch pipettes had a resistance of 5–6 MΩ filled with the intracellular solution containing 130 mM K-gluconate, 1 mM CaCl_2_, 1 mM MgCl_2_, 10 mM EGTA, 10 mM sucrose and 20 mM HEPES (pH adjusted with KOH, 305 mOsm). In voltage-clamp recordings, differentiated cells were held at −80 mV and voltage pulses (10 mV/step, 30 msec) were applied to elicit voltage-activated currents. Action potentials were evoked by injecting currents (20 pA/step, 300 msec) at −80 mV in a current-clamp mode. Data were digitized at 10 kHz with a 2 kHz low-pass filter. Liquid junction potential was corrected.

### Calcium measurement assays

Neurons derived from iPSCs transfected with syn-5TFs mRNAs were grown on glass cover slips and used for calcium imaging at Day7. Cells were loaded with 1.5 μM Fluo-4 AM (Thermo Fisher Scientific, Waltham, MA) in a buffer consisting of 117 mM NaCl, 2.5 mM KCl, 2 mM CaCl_2_, 2 mM MgSO_4_, 25 mM HEPES (pH 7.4) and 30 mM d-(+)-glucose for 20 min at 37 °C. After washing the dye with the buffer for 30 min, the coverslips were placed in a chamber equipped with electrodes for field stimulation. Cells were perfused with the buffer at room temperature with or without 0.5 μM tetrodotoxin (TTX, Alomone Labs, Israel). Images were captured at 2 Hz (exposure time of 500 ms) with a Nikon Eclipse microscope using a 20x objective equipped with a CCD camera (Andor iXon, DU897). For extracellular electric field stimulation, 40 Hz of 500 μs pulses were applied for 5 s. Images were analyzed using ImageJ software (Rasband, W.S., ImageJ, U. S. National Institutes of Health, Bethesda, Maryland, USA, http://imagej.nih.gov/ij/, 1997–2015.)

### RNA seq analysis

SEES3 human ES cells were transfected for a total of four times over two days with either Emerald (two times on Day 1) or mCherry (two times on Day 2) and Neurogenin3 synthetic mRNA (total of four times, two times in a day). Forty-eight hours after the start of the first transfection, the cells were harvested and lysed with Trizol reagent, and total RNA was extracted and was analyzed for RNA seq analysis.

### Scoring marker-positive cells and statistical analysis

To assess the efficiency of cell differentiation, photographs obtained by the immunocytochemistry with various differentiation markers were visually inspected and the number of marker-positive cells were counted (e.g., Figs 3a,c,d and 6a). For each experiment, a total number of marker-positive cells were obtained by summing the number of marker-positive cells from 3 to 5 photographs captured from different fields of immunocytochemistry. For statistical analyses, a total number of marker-positive cells obtained from at least two independent differentiation experiments were used to calculate the mean and standard deviation (SD).

### Quantitative PCR analysis

Quantitative RT-PCR (qPCR) was carried out using cDNAs obtained from undifferentiated or syn-5TFs-treated TkDA3-4 human iPS cells at various time points. qPCR was performed using the SYBR Premix ExTaq II (Takara Clonetech) and TAKARA CyclerDice Real Time System II (Takara, Kusatsu, Shiga, Japan). GAPDH was used as a reference gene and the relative gene expression levels were obtained by normalizing with GAPDH expression.

## Additional Information

**How to cite this article**: Goparaju, S. K. *et al*. Rapid differentiation of human pluripotent stem cells into functional neurons by mRNAs encoding transcription factors. *Sci. Rep.* **7**, 42367; doi: 10.1038/srep42367 (2017).

**Publisher's note:** Springer Nature remains neutral with regard to jurisdictional claims in published maps and institutional affiliations.

## Supplementary Material

Supplementary Movie M1

Supplementary Information
