# Supplementary material for: Rapid differentiation of human pluripotent stem cells into functional neurons by mRNAs encoding transcription factors
Source: Sci Rep. 2017 Feb 13;7:42367. doi: 10.1038/srep42367 (PMC5304326; doi:10.1038/srep42367)
Supplement: Supplementary Information [file srep42367-s2.pdf]

Supplementary Information

Rapid differentiation of human pluripotent stem cells into functional motor neurons by mRNAs encoding transcription factors

Sravan Kumar Goparaju, Kazuhisa Kohda, Keiji Ibata, Atsumi Soma, Yukhi Nakatake, Tomohiko Akiyama, Shunichi Wakabayashi, Misako Matsushita, Miki Sakota, Hiromi Kimura, Michisuke Yuzaki, Shigeru B.H. Ko and Minoru S.H. Ko

Supplementary tables – Table S1 provides the electrophysiological characteristics of syn-5TFs-derived neurons (day10) from TkDA3-4 iPS cells**; Table S2** provides the list of primers used for Reverse Transcription-PCR and Tail-PCR; **Table S3** provides the list of antibodies, their sources, and dilutions used.

Supplementary Figures – Fig. S1 shows the kinetics of NGN2 protein expression in TkDA3-4 iPS cells ectopically expressing syn-NGN2 mRNA; **Fig. S2** shows the induction of neurogenesis by syn-TF mRNAs of Neurogenins and NeuroD families in human ES cells; **Fig. S3a-b** show the original blots of time course of NGN2 (and β-actin loading control) protein expression in lysates of human ES cells transfected with syn-NGN2 mRNA (corresponding to Figure 1c); **Fig. S4a-b** show the original blots of time course of Emerald (and β-actin loading control) protein expression in lysates of human ES cells transfected with syn-Emerald mRNA (corresponding to Figure 1d); **Fig. S5.** qPCR analyses of motor neuron markers in human iPS-derived neurons.

Supplementary Movie M1 depicts the calcium transients in neuronal cells derived from iPS cells.

Supplementary Table S1:

Electrophysiological characteristics of syn-5TFs-derived neurons (Day10) from TkDA3-4 iPS cells

	# of action potentials				Resting membrane potential (mV)	Rm (MΩ)	Capacitance (pF)
	total	0	1	2 ≦			
n (%)	35 (100)	2 (5.7)	12 (34.3)	21 (60.0)	-33.0 ± 2.4	516 ± 48	14.4 ± 1.4

Data represent mean ± SEM.

Supplementary Table S2:

Primers used in this study:

For RT-PCR

Gene	Forward Primer (5' – 3')	Reverse Primer (5' – 3')
GAPDH	GGTGGTCTCCTCTGA<br>CTTCAACA	GTGGTCGTTGAGGGCAATG
ChAT	ACTGGGTGTCTGAGT<br>ACTGG	TTGGAAGCCATTTTGACTAT
HB9	CCTAAGATGCCCGA<br>CTTCAACTC	GCCTTTTTGCTGCGTTTCCATTTC
Tail PCR	TAATACGACTCACTA<br>TAGGG<br>TTGGACCCTCGTACA<br>GAAGCT	TTTTTTTTTTTTTTTTTTTTTTTTTTTT<br>TTTTTTTTTTTTTTTTTTTTTTTTTTTTTTTTTTTTTT<br>TTTTTTTTTTTTTTTTTTTTTTTTTTTTTTTTTTTTTT<br>TTTTTTTTTTTTTTTGCGTCGACACTAGTTCTAGA<br>CCCTCACTTC

Primers for QPCR

Gene	Forward Primer	Reverse Primer
ISL1	CAGGTTGTACGGGATCAAATGC	CACACAGCGGAAACACTCGAT
CHT1	AAGCCATCATAGTTGGTGGCCGAG	AAGCCATCATAGTTGGTGGCCGAG
HB9	GCACCAGTTCAAGCTCAACA	TTTGCTGCGTTTCCATTTC
ChAT	TCATTAATTTCCGCCGTCTC	GAGTCCCGGTTGGTGGAGT

Supplementary Table S3:

Antibodies used in this study:

**Antibody**	**Source**	**Catalog #**	**Dilution**	**Species**
β-actin (13E5)	CST	4970	1:2000	Rabbit
βIII-tubulin neuron spec. SDL.3D10	Sigma	T8660	1:1500	Mouse
βIII-tubulin	CST	5568	1:1000	Rabbit
ChAT	Millipore	AB143	1:1000	Rabbit
E-Cadherin (24E10)	CST	3195	1:500	Rabbit
GFP (mix of 7.1 and 13.1 clones)	Roche	11814460001	1:1000	Mouse
HB9	DSHB	81.5C10-s	1:50	Mouse
ISL1	Abcam	20670	1:1000	Rabbit
Ki67	CST	9129	1:1000	Rabbit
MAP2	Sigma	HM-2	1:1000	Mouse
NeuN, Clone A60	Millipore	MAB377	1:1000	Mouse
NEUROGENIN2	CST	13144	1:1000	Rabbit
POU5F1 (OCT3/4)	SCBT	sc-5279	1:1000	Mouse

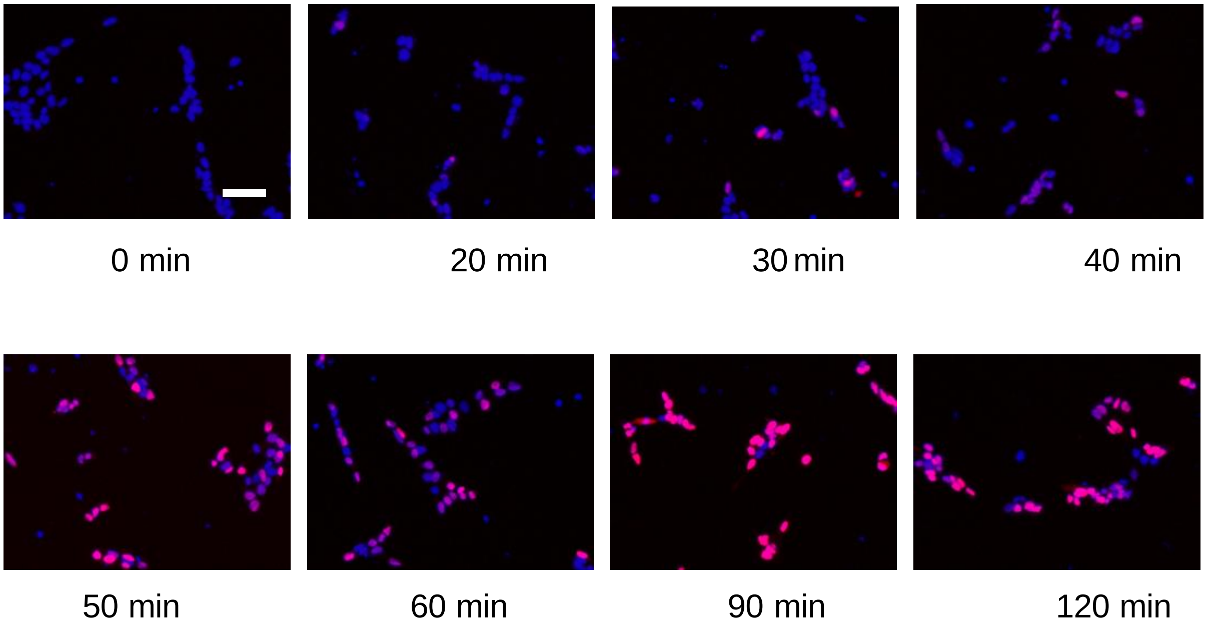

Supplementary Figure S1. Kinetics of NGN2 protein expression in TkDA3-4 iPS cells ectopically expressing syn-NGN2 mRNA. NGN2 protein expression was determined by using an antibody against NGN2. Nuclei (blue); NGN2 (pink). Scale bar indicates 200 µm.

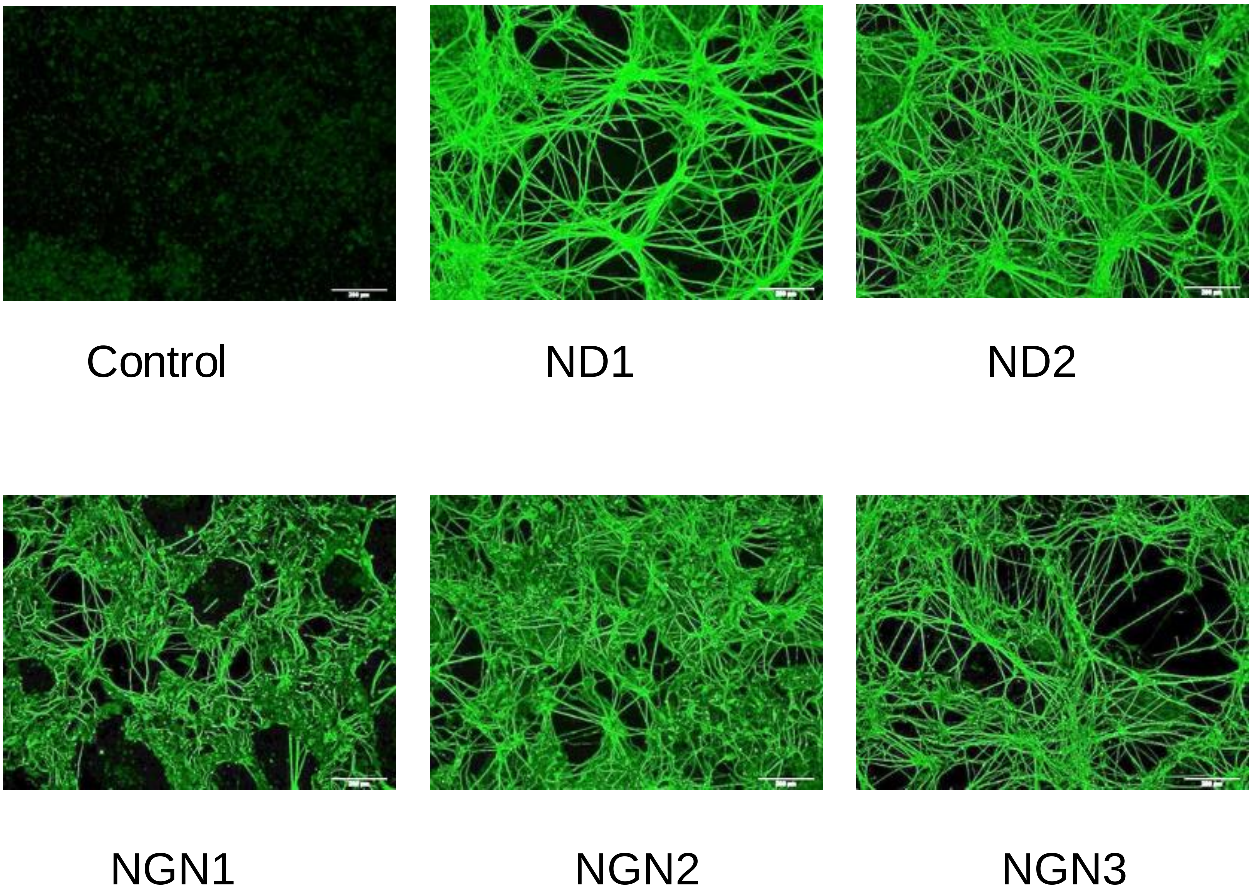

Supplementary Figure S2. Induction of neurogenesis by syn-TF mRNAs of Neurogenins and NeuroD families in human ES cells. Neuronal differentiation induced by syn-mRNAs encoding NeuroD1 (ND1), NeuroD2 (ND2), Neurogenin1 (NGN1), Neurogenin2 (NGN2), Neurogenin3 (NGN3) in SEES3 human ES cells at Day 7. Expression of neuron-specific βIII-tubulin (TUBB3) was measured by immunocytochemistry. Scale bars indicate 200 µm.

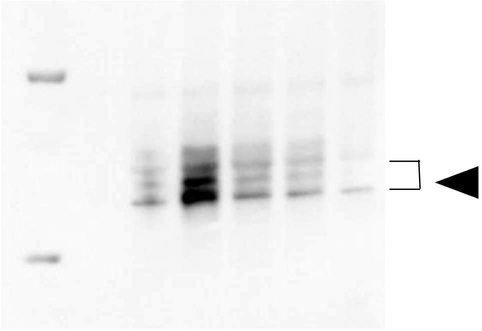

M 0 8 16 24 32 hours

Supplementary Figure S3a. Original blot of time course of NGN2 protein expression in lysates of human ES cells transfected with syn-NGN2 mRNA (corresponding to upper panel of Figure 1c). M indicates molecular weight markers. NGN2 protein is indicated by an arrowhead.

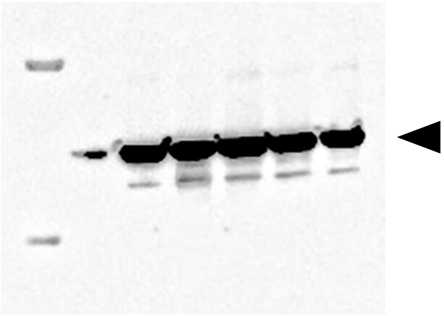

M 0 8 16 24 32 hours

Supplementary Figure S3b. Original blot of time course of β-actin protein expression (used as a loading control) in lysates of human ES cells transfected with syn-NGN2 mRNA (corresponding to upper panel of Figure 1c). Stripped blot in Supplementary figure S3a was re-probed with an antibody against β-actin Left most lane shows molecular weight markers. β-actin protein is indicated by an arrowhead M indicates molecular weight markers. β-actin protein is indicated by an arrowhead.

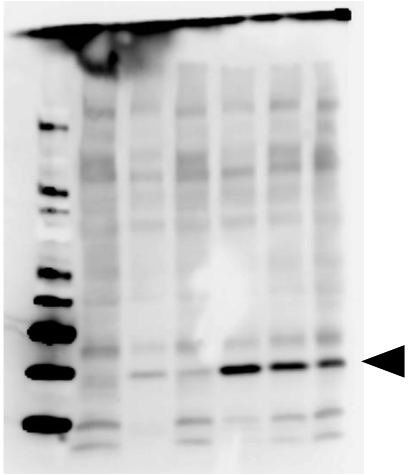

M 0 6 12 18 24 48 hours

Supplementary Figure S4a. Original blot of time course of Emerald protein expression in lysates of human ES cells transfected with syn-Emerald mRNA (corresponding to upper panel of Figure 1d). M indicates molecular weight markers. Emerald protein is indicated by an arrowhead.

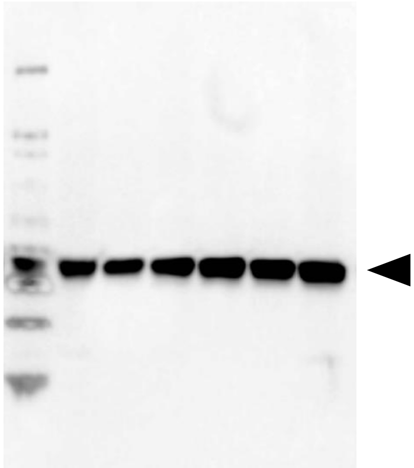

M 0 6 12 18 24 48 hours

Supplementary Figure S4b. Original blot of time course of β-actin protein expression (used as a loading control) in lysates of human ES cells transfected with syn-Emerald mRNA (corresponding to lower panel of Figure 1d). Stripped blot in Supplementary figure S4a was re-probed with an antibody against β-actin Left most lane shows molecular weight markers. β-actin protein is indicated by an arrowhead.

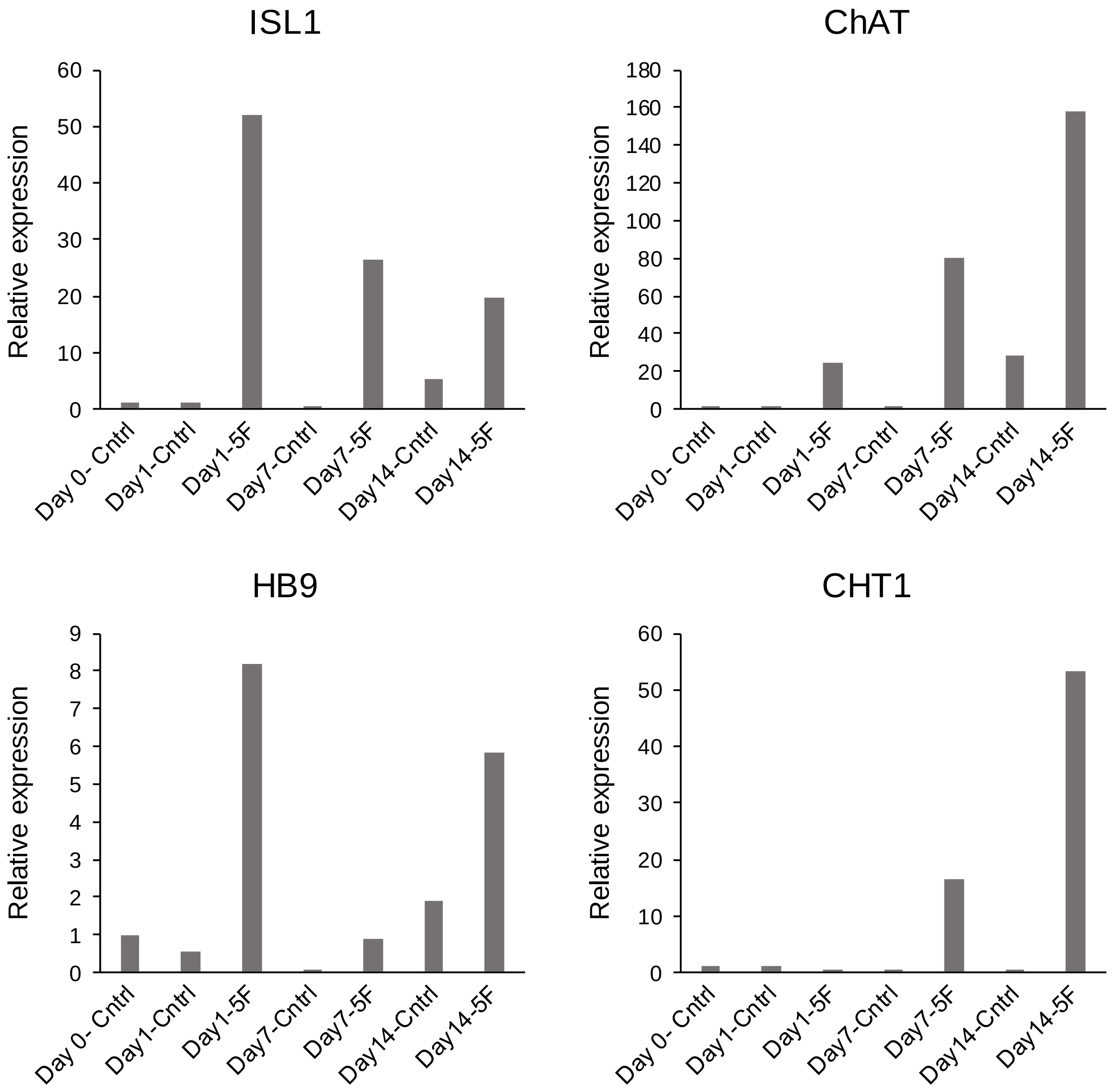

Supplementary Figure S5. Quantitative RT-PCR (qPCR) analyses of motor neuron markers in human iPS-derived neurons. qPCR was carried out using cDNAs obtained from undifferentiated or syn-5TFs differentiated TkDA3-4 human iPS cells at various time points. GAPDH was used as a reference gene and the mean of duplicate samples normalized to GAPDH expression was calculated. Relative expression to control TkDA3-4 human iPS cells (Day 0) is shown.

Supplementary movie M1

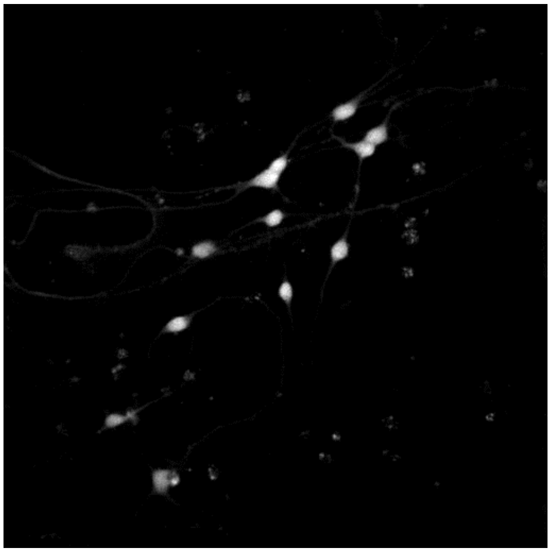

Supplementary Movie M1. Movie depicting the calcium transients in syn-5TFs cocktail-induced neuronal cells (Day 7). Fluo-4 loaded cells stimulated with 40 Hz pulse. Uploaded as a separate Movie file.
